# Only a Dentist

**Published:** 1921-08

**Authors:** 


### "Only a Dentist"

Once upon a time, at a certain golf club, of which I am a member, I was introduced to a "Doctor X."

I found him a likable fellow—which is saying something—for he walloped me at golf, and being walloped at golf seldom prejudices you in favor of the walloper.

The next day I met Doctor Z., whom I had known for some time and who belonged to the same club. I mentioned having been introduced to Doctor X., and what a good fellow I had found him to be. Doctor Z. thereupon took particular pains to inform me that Doctor X. was not a "real doctor," but "only a dentist."

Why the distinction, is beyond me.

The word "doctor" means master, in the sense that the one bearing the title is a master of some branch of learning. The original meaning of the word "doctor" was that its bearer was a teacher, an instructor, a learned man, one skilled in some learned profession.

There were doctors of laws, doctors of languages, doctors of divinity, as well as doctors of philosophy, long before the practice of medicine was recognized.

Nevertheless, along comes the medical "profesh"—almost the last on the list—usurps to itself the title of doctor and relegates to the rear all other claimants. The result of this effrontery is that the wearer of the title of doctor who isn't a medical practitioner is looked at somewhat askance, as if he had no right to it.

This arrogance on the part of the medico is all the more astounding in the light of the fact that the practice of medicine is not an exact science, but actually a sort of "catch-as-catch-can" science.

But the medicos parade themselves as exact scientists, nevertheless, though their pretensions are ridiculous. Why, if they were what they would have us believe them to be, measles, scarlet fever, chicken pox, whooping cough, mumps and all those kindred ills which almost every kiddie has to fall heir to would have been wiped out of existence long ago.

The surgeon has more claim to being an exact scientist than the medico. Surgery has advanced to a wonderful state of accuracy and precision; in fact, surgery never did surround itself with mystery and has nearly always been ready to frankly admit its ignorance when it didn't know.

Surgery has been ruthless, it is true, in its acquirement of knowledge, and has slaughtered lives, both human and animal, by the thousands in order to learn, but it has not been guilty of the chicanery and mysticism by which the medical profession has covered up its ignorance.

The surgeon is a direct actionist. He cures by the employment of radical measures, but he is also the servant of the Greatest Physician in the World—Nature—in that he removes the obstacles which prevent Nature from applying her healing treatment.

Nature cures through the circulation of the blood. A part of the anatomy of a human being may be so diseased or injured that Nature's efforts to restore a perfect circulation, and thus effect her curative measures, are baffled. In steps the surgeon with his scalpel, and severs or separates the diseased or injured part, thus removing the obstacle to Nature's restoration of the blood circulation. Then Nature completes the cure by applying her remedies through the blood and by removing, through the blood, the diseases tissues which the surgeon's knife has not eliminated.

The practice of dentistry is merely a branch of surgery and the dentist is in fact a "dental surgeon."

The doctor of dental surgery can guarantee results in nine cases out of ten.

Instead of trying to know all the human anatomy, he has concentrated on one specific portion, and as a result his cures are, with rare exceptions, certain.

Furthermore, the dentist can supply artificial bones for those he removes, which artificial bones are in many cases an improvement on the ones supplied by Nature, In this respect, he rather surpasses the surgeon, whose replacement ability has yet hardly gone beyond the stage of experimentation.

A dentist is also one of the greatest preventers of disease, for many diseases are created by bad teeth, or at least by poor digestion due to bad teeth.

From early boyhood, my wise parents sent me to the dentist once every six months. Since accumulating a fraction of their wisdom in my own cranium, I have continued this practice and I have kept all my teeth except one and have had only two filled, though I'm some years past fifty.

The one I lost I sacrificed that I might be in condition to make a speech. It was either no tooth or no speech, so I let it go, but I have often since wished I had decided the other way about. In those days, however, I hadn't yet become a Near-Editor and was not invited to make so many speeches as I am now.

"Only a dentist!"

If a dentist was no more certain of his profession than the medical practitioner, he would be petitioning the law-making bodies to pass laws prohibiting folks from pulling their own teeth.

In that case, my grandson might be doing time for malpractice, for he has pulled his sister's teeth by the simple process of attaching one end of a string to the tooth, the other end to a door knob, and then slamming the door.

My grand-kiddie has practiced for pay, too, for he received a cash consideration for each operation, and then divided fifty-fifty with his patient, an act of generosity I believe unheard of in the medical profession.

I believe that the dentists have given modern civilization as many benefits as the medical men have, and I believe that they have done it with the expenditure of far less "bull."

And the more I ponder on the fact that Doctor X. is "only a dentist," the more I like him and admire him and the less I like Doctor Z. Perhaps it is because Doctor X. *is* only a dentist.—*The Houghton Line,* May, 1921.

